# Laboratory-based turning performance during walking in people with mild cognitive impairment and dementia

**DOI:** 10.1177/13872877261450969

**Published:** 2026-05-27

**Authors:** Ríona Mc Ardle, Andrew Kingston, Silvia Del Din, Rana Zia Ur Rehman, Brook Galna, Alan J. Thomas, Lynn Rochester, Lisa Alcock

**Affiliations:** 1 Translational and Clinical Research Institute, Faculty of Medical Sciences, 5994 Newcastle University, Newcastle Upon Tyne, UK; 2 NIHR Newcastle Biomedical Research Centre, 5994 Newcastle University and The Newcastle upon Tyne Hospitals NHS Foundation Trust, Newcastle upon Tyne, UK; 3 Population Health Sciences Institute, Faculty of Medical Sciences, Newcastle University, Newcastle Upon Tyne, UK; 4 Janssen Research & Development, High Wycombe, UK; 5 School of Allied Health (Exercise Science), 5673 Murdoch University, Perth, Western Australia, Australia; 6 Centre for Healthy Ageing, Health Futures Institute, 5673 Murdoch University, Perth, Western Australia, Australia

**Keywords:** Alzheimer's disease, cognitive impairment, dementia, gait, turning, wearable technology

## Abstract

**Background:**

Mobility impairments during straight-line walking show utility in detecting dementia-related cognitive impairment (e.g., Alzheimer's disease). Wearable technologies can now derive mobility outcomes related to turning, considered a more cognitively complex movement. However, it is unclear which turning variables would best detect cognitive impairment.

**Objective:**

This study aimed to develop a data-driven model of turning performance relevant to cognitive impairment and compare selected turning variables between people with cognitive impairment and cognitively-intact older adults.

**Methods:**

77 people with cognitive impairment (31 mild cognitive impairment, 46 dementia) and 28 cognitively-intact older adults. performed six intermittent walking trials with a 180° turn while wearing an inertial measurement unit (APDM Opal) attached to their lower back. 102 spatiotemporal and signal-based turning variables were captured. Following data reduction, 15 variables underwent exploratory factor analysis. One-way ANCOVA, adjusting for age, sex and height, explored between-group differences (Bonferroni correction applied).

**Results:**

Three factors emerged: turn initiation, magnitude and smoothness, accounting for 67% of the total variance in turning performance. The cognitively impaired group demonstrated significant differences in turn initiation (start-phase root mean square (RMS) in vertical direction, start-phase jerk RMS in vertical/mediolateral/anterior-posterior directions) and magnitude (mid-phase RMS in mediolateral direction, mean RMS in vertical direction) domains (p < 0.003) with moderate-large effect sizes (Partial η^2^ = 0.09–0.17). Turn initiation and magnitude factor scores were associated with cognitive performance.

**Conclusions:**

Key findings suggest that people with cognitive impairment exhibit smaller and slower turning movements specifically during turn initiation and demonstrate lower signal magnitude (RMS) across the turn. Further work should consider use of these variables in predicting incidence of cognitive impairment in at-risk populations.

## Introduction

Dementia is a progressive neurological condition, associated with significant impairments across multiple cognitive domains which impact one's ability to carry out activities of daily living.^1^ Although mobility impairments (e.g., gait, balance) are often an under-recognized feature of dementia disease progression, ample evidence indicates that gait impairments occur years prior to onset of cognitive symptoms.^2–4^ Growing research suggests that spatiotemporal gait outcomes, captured through motion analysis, instrumented walkways, and wearable technologies, may aid in the prediction and detection of cognitive impairment.^4–9^ Emerging evidence also demonstrates that different dementia subtypes may have unique signatures of gait impairment when undergoing straight-walking clinical assessments, with gait proposed as a potential supportive marker within the clinician's differential diagnosis toolkit.^10–12^ Characterizing mobility impairments accurately is paramount to developing and validating appropriate physical and digital markers of cognitive decline and disease subtype.

Recently, there has been a growing interest in the relationship between turning performance during gait activities and cognitive function. Turning impairments, such as slower turns, have been associated with greater executive dysfunction, attentional and visuospatial impairments and slower perceptual speed in people with cognitive impairment and Parkinson's disease, reflecting findings in straight-line gait.^13–16^ Turning is more complex and cognitively demanding than straight-walking as it requires considerable planning and multi-segment co-ordination to execute.^13,17–19^ Turning performance may therefore be impaired in people with cognitive impairment due to dementia-causing diseases such as Alzheimer's disease, Lewy body disease, and vascular dementia, as shown with straight-line gait, possibly supporting early detection of these conditions.^5,9–12^ However, there has been limited research in this area with only three studies using diverse cognitively-impaired groups (mild cognitive impairment (MCI), overall cognitive impairment and long-term care residents) resulting in conflicting findings (longer and shorter turn duration respectively) compared to community-dwelling older adults.^15,20,21^ Further research is required to investigate turning performance in people with cognitive impairment using a well-characterized sample to understand their potential use in detecting cognitive impairment.

Inertial measurement units (IMUs) allow detection of mobility-related outcomes beyond straight-walking, such as turning performance.^20,22–25^ This is because a single IMU worn on the lower back can capture angular rotations related to whole body movement and center of mass using the gyroscope signal.^24^ There are multiple ways to characterize turning performance using spatiotemporal, kinematic and signal-based variables,^24^ with limited consensus on which variables are most relevant or clinically-meaningful to those with cognitive impairment. The lack of standardization and abundance of possible variables creates challenges for interpreting findings, comparing across studies and translating research into clinically applicable outcomes. Recently, an IMU-based turn detection algorithm was validated for use in people with cognitive impairment while turning ≥ 90° during an intermittent walking task.^25^ This algorithm can derive over one hundred turning variables, highlighting the need for a clear framework to identify the most relevant outcomes for cognitive impairment. A potential approach would be to group turning variables into independent domains via factor analysis, as commonly reported in gait studies in neurological conditions.^26,27^

Therefore, the aims of this study were to (1) generate a model of turning performance specific to people with cognitive impairment (inclusive of MCI and dementia) through exploratory factor analysis, and (2) to identify differences in selected turning variables and factors between people with cognitive impairment and cognitively-intact older adults. We hypothesized that people with cognitive impairment would demonstrate greater impairments in turning performance compared to cognitively-intact older adults.

## Methods

### Participants

This is a secondary analysis of the GaitDem study, previously described.^10,11,25^ Participants with probable MCI or dementia due to AD, Lewy body disease (i.e., dementia with Lewy bodies or Parkinson's disease dementia) or vascular dementia were recruited from relevant clinical services in North East England and the Dementia and Neurodegenerative Diseases Research Network (DeNDRoN). Standardized diagnostic criteria were used to define diagnosis of dementia subtype, with consensus clinical review for verification.^28–34^ This included formal diagnostic criteria for Alzheimer's disease,^28^ dementia with Lewy bodies,^29^ Parkinson's disease dementia^31^ and vascular dementia.,^34^ and standardized clinical or research criteria for MCI, involving consideration of the underlying disease pathology such as Alzheimer's disease,^33^ dementia with Lewy bodies,^30^ and Parkinson's disease.^32^ Cognitively-intact older adults were recruited via DeNDRoN.

Eligibility criteria for both groups were defined as follows. To be included, participants must be ≥60 years, able to walk for two-minutes (self-report), have a good command of the English language, and have the capacity to consent. Participants with cognitive impairment required a formal diagnosis of MCI or dementia due to Alzheimer's disease, dementia with Lewy bodies, Parkinson's disease, or vascular dementia. Cognitively-intact older adults were included only if they showed no evidence of cognitive impairment (Mini–Mental State Examination (MMSE) ≥25) and were functionally independent. Exclusion criteria for all participants included evidence of drug-induced/vascular parkinsonism, co-existing neurological conditions or movement disorders except Parkinson's disease-related MCI or dementia, severe mental illness, or evidence of stroke severely affecting motor function. All participants with Parkinson's disease MCI or dementia were taking Parkinson's disease-related medication (e.g., levodopa) and underwent gait assessment while ON their medication.

### Demographic and clinical measures

Demographic information was collected, including age, sex and height. Premorbid IQ was assessed using the National Adult Reading Test. Co-morbidities were calculated using the Cumulative Illness Rating Scale – Geriatrics. Motor disease severity was assessed using the Movement Disorders Society Unified Parkinson's Disease Rating Scale Part III.^35^

### Cognitive measures

Global cognition was measured using both the standardized MMSE (sMMSE) and the Addenbrookes Cognitive Examination III total scores (ACE-III). Relevant to this analysis, visuospatial abilities were assessed using the ACE-III visuospatial subscale, while information processing speed was measured using the Trail Making Task Part A (TMT A). Executive function was assessed via the FAS verbal phonemic fluency test. Attention was measured using the simple reaction time computerized test.^36^

### Turning performance during laboratory-based walking protocol

Participants were asked to complete six intermittent walks across a 10m walkway at a self-selected comfortable pace while wearing a tri-axial inertial measurement unit (APDM, Opal 128 Hz, Inc., Portland, OR, USA), consisting of a triaxial accelerometer (range: ±6 g), gyroscope (range: ±2000 degrees per second (dps)) and magnetometer (range: ± 6 Gauss), on their lower back (5th lumbar spine) secured in place with an elasticated Velcro strap. The start and end of each walk was demarcated by a line of tape on the floor. All participants were provided the opportunity to complete a practice walk before the assessment began. For all walks, they were asked to walk to the line at the end and to turn around (∼180-degree turn). Turn direction or strategy was not stipulated. They were then asked to wait until they were instructed to walk back to the other line and turn around. Data collection was conducted using a standardized operating procedure to ensure assessment consistency, and all assessors adhered to identical written procedures. During every assessment, at least one of two key researchers (RMA, LA) were present, reducing inconsistencies between participant testing sessions. No participants used a walking aid to complete the gait assessment.

### Data processing for turn detection

Signal data were processed using a validated algorithm for evaluating 180 degree turns.^25^ The pseudocode has been published elsewhere.^24^ Acceleration and gyroscope data were detrended and filtered using a 4th order low-pass Butterworth filter and cut-off frequency of 20 Hz. A turn was defined as a rotation of the lower back IMU ≥ 90 degrees about the vertical axis with a minimum and maximum turn duration set (0.5–10.0 s). Turns ≥ 10 degrees and ≤0.5 s in the same direction were merged and considered as a single turn. Biases and noise associated with the gyroscope signal were removed using a compensation algorithm. Zero crossing of the vertical angular velocity was used to define the turn start and end.^24^

Turning features were extracted for each of the five turns and the mean was used in subsequent analysis.

Spatiotemporal variables included turn duration (seconds), angular velocity and peak angular velocity (degrees/second). Signal based variables included root mean square (i.e., the magnitude movement recorded from both the accelerometer and gyroscope signals), jerk (i.e., rate of change of acceleration), and jerk root mean square (RMS; i.e., how consistently and rapidly the magnitude of acceleration changes over time) in the mediolateral, anterior-anterior posterior, vertical and combined directions. All variables were considered by their mean, minimum, maximum, range, start-phase, mid-phase and end-phase components. Variability (SD) of spatiotemporal variables was also derived; however, these were removed prior to analysis as five turns is not suitable for a reliable calculation of variability.

### Statistical analysis

Statistical analysis was divided into three phases: Data reduction and exploratory factor analysis (EFA) to create a model of turning performance in our sample of people with cognitive impairment; Exploration of between-group differences (cognitive impairment versus cognitively-intact older adults) in final turning variables and factors; and Correlations between cognitive domains and turning factors.

#### Data reduction and exploratory factor analysis

Data reduction involved the use of Spearman's rank correlations between turning variables and cognitive scores on the whole dataset to identify candidate variables for the EFA. Associations between candidate turning variables were then explored using Spearman's rank correlations, with correlation coefficients ≥ 0.30 but <0.90 retained.^37,38^ Kaiser-Meyer-Olkin (KMO) values for each single variable of >0.50 and overall KMO of >0.70 were considered acceptable for analysis.^39^ EFA was conducted using principal components as the extraction method. Number of factors was determined as those with an eigenvalue > 1,^40^ with Cattell's Scree Test and Horn's parallel analysis supporting selection. A Promax oblique rotation method was used.^38^ Primary loadings were retained if the value was ≥0.50 and the difference between the highest and second-highest absolute loadings was ≥0.20, to minimize effects of cross-loading.^38^ Further details on the data reduction and EFA analysis can be found in the Supplemental Material. Given the inclusion of participants with Parkinson's disease cognitive impairment, who may exhibit motor-related turning impairments, a sensitivity analysis was performed by excluding these participants (n = 10) from the sample and re-running the exploratory factor analysis.

#### Between-group differences

For the primary analyses, participants with a formal clinical diagnosis of MCI or mild dementia were combined into a single cognitive impairment group to reflect the clinical continuum of cognitive impairment and to increase statistical power for the detection of mobility differences relative to cognitively intact older adults. Between-group differences (cognitively impaired versus cognitively-intact older adults) for demographic and clinical characteristics were assessed using Pearson's chi-square test for categorical variables and Mann Whitney U test for continuous variables. Differences between groups in turning variables and factors were examined using one-way ANCOVA, adjusting for age, sex, and height which are common covariates in the gait literature. Histograms were inspected and Shapiro Wilk tests inspected distribution of residuals for each model. Where data were positively skewed, log10 transformation or square-root transformation was applied, depending on degree and nature of skewness, while negatively-skewed data were reflected and then log10 transformed (y = log10(K-x) with K = max(x) + 1). A Bonferroni-adjusted significance threshold was applied to account for multiple comparisons across the included variables. Sensitivity analyses were performed by (i) excluding participants with Parkinson's disease cognitive impairment and re-running the ANCOVA to account for potential motor-related effects, and (ii) examining differences between MCI and dementia subgroups to confirm the appropriateness of combining these groups for analysis.

Partial eta squared (η^2^) was calculated as a measure of effect size, with values of 0.01 interpreted as small, 0.06 as medium and 0.14 as large.^37^ Partial η^2^ CIs were wide, with upper bounds at 1.00 for all outcomes, reflecting limited information (unbalanced groups); therefore only point estimates are reported, with effect sizes interpreted cautiously.

#### Associations between turning factors and cognitive variables

Exploratory associations between the produced turning factors and cognitive scores relating to global cognition, attention, information processing, executive function and visuospatial ability were examined using Spearman's rank correlations in the whole dataset (including cognitively-intact older adults). This was to determine if the produced factors were meaningfully associated with cognitive performance.

## Results

Out of the 125 participants recruited to the GaitDem study, 105 participants were included in this analysis. Reasons for data loss include participant withdrawal (n = 2), exclusion due to differential diagnosis other than MCI or dementia (n = 4) and missing IMU data due to technical failure (n = 14). Participants included 77 people with cognitive impairment (MCI: 31, Dementia: 46) and 28 cognitively-intact older adults (see Table 1 for further details; see Supplemental Tables 1 and 2 for breakdown of groups by dementia severity and subtype).

**Table 1.** Demographic and clinical information for participants.

	N	Cognitive impairment N = 77	Cognitively-intact older adults N = 28	p
Cognitive severity	105			
MCI		31 (40%)		
Dementia		46 (60%)		
Subtype	105			
Alzheimer's disease		35 (45%)		
Dementia with Lewy Bodies		25 (32%)		
Parkinson's disease cognitive impairment		10 (13%)		
Vascular dementia		7 (9.1%)		
Age (years)	105	76 (65–91)	73 (60–89)	0.10
Sex (male)	105	50 (65%)	11 (39%)	**0**.**018**
Height (m)	105	1.69 (1.45–1.87)	1.67 (1.52–1.83)	0.4
MDS-UPDRS III (n/132)	101	15 (0–70)	1 (0–11)	**<0**.**001**
sMMSE (n/30)	105	24 (14 −30)	30 (25–30)	**<0**.**001**
ACE-III Total (n/100)	104	75 (15–95)	97 (87–100)	**<0**.**001**
ACE-III VS (n/16)	104	14 (0–16)	16 (13–16)	**<0**.**001**
FAS Total	102	30 (7–61)	45 (29–69)	**<0**.**001**
TMT A (secs)	94	60 (24–955)	30 (19–65)	**<0**.**001**
Simple RT (secs)	99	442 (287–3792)	372 (291–493)	**<0**.**001**
CIRSG (n/56)	104	9 (3–19)	4 (0–11)	**<0**.**001**
NART	103	116 (100–125)	123 (114–126)	**<0**.**001**
Taking Parkinson's medication (% Yes)	105	13 (17%)	0 (0%)	**0**.**018**
Taking anti-dementia medication (% Yes)	98	48 (69%)	0 (0%)	**<.0.001**

N(%), median (range); MCI: mild cognitive impairment; UPDRS-III: Movement Disorders Society Unified Parkinson's disease rating scale; sMMSE: standardized Mini Mental State Examination; ACE-III: Addenbrooke's Cognitive Examination; VS: visuospatial; FAS: FAS verbal fluency test; TMT A: Trail making test A; RT: reaction time; CIRSG: Cumulative illness rating scale – geriatric; NART: National Adult Reading Test (IQ).

### Associations between cognitive domains and turning variables

Associations between five cognitive scores and 95 turning variables were initially considered, with 33 turning variables retained for subsequent analysis based on a Spearman Rho correlation ≥ 0.3 with at least one cognitive score (see Supplemental Figures 1 and 2). Associations between retained turning variables were examined; after excluding those showing redundancy, 23 variables were taken forward to EFA (see the Supplemental Material and Supplemental Figure 2 for further detail).

### Exploratory factor analysis

**Figure 1.**
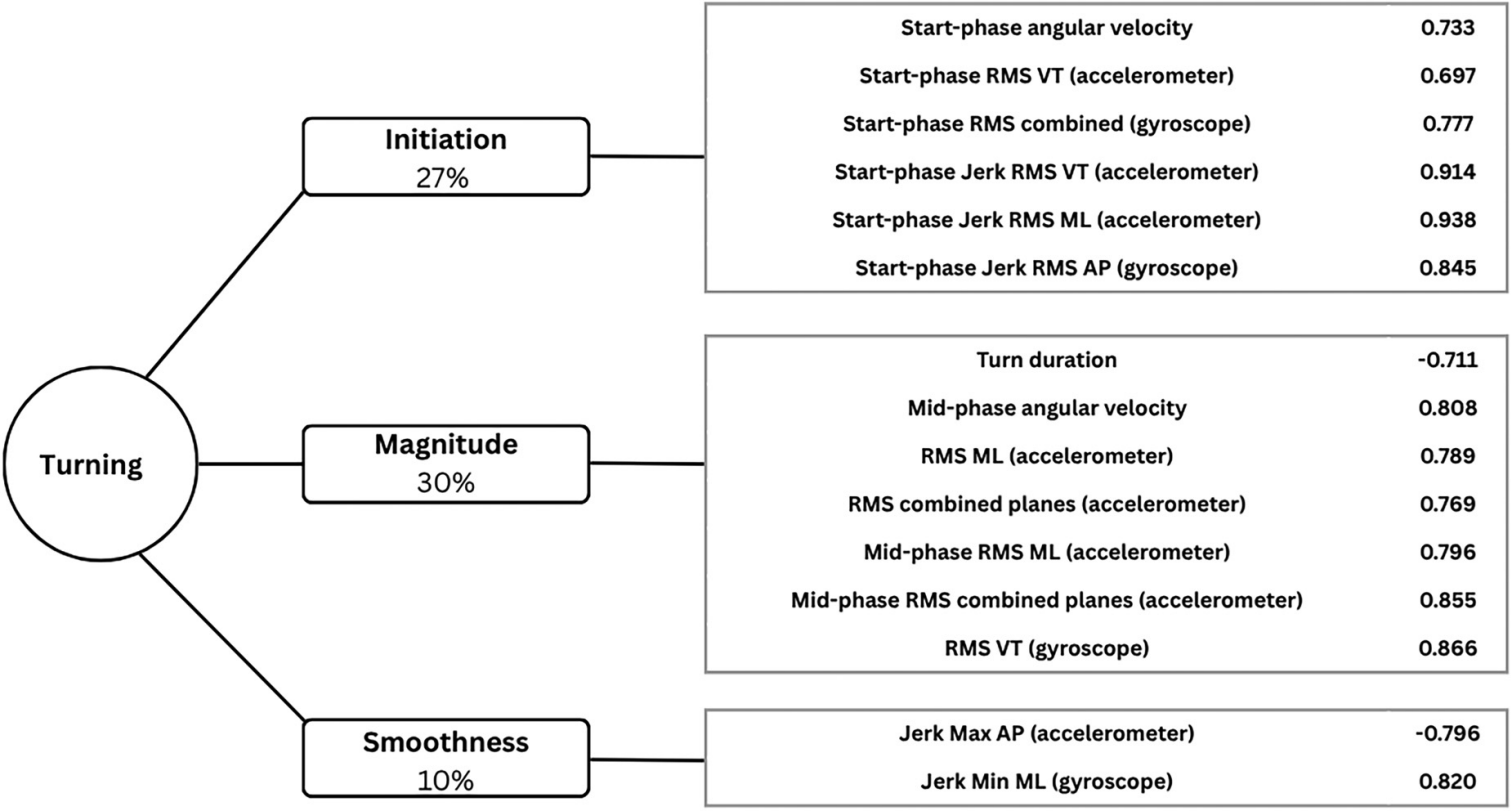
A conceptual model of turning performance for people with cognitive impairment. The values in the boxes represent the proportion of total variance explained by each domain. Factor loadings provided beside variable names. RMS: root mean square; VT: vertical axis; ML: mediolateral; AP: anterior-posterior.

Full details of the EFA can be found in the Supplemental Material. Three factors across 15 turning variables were revealed, which accounted for 67% of the total variance in turning performance (Turn Initiation: 27%, Turn Magnitude: 30%, Turn Smoothness: 10%). Factor loadings are reported in Figure 1. Our sensitivity analysis, whereby we removed participants with Parkinson's disease cognitive impairment, revealed a highly consistent structure compared to the primary analysis reported here (see the Supplemental Material and Supplemental Table 3).

### Between-group differences for turning variables

**Figure 2.**
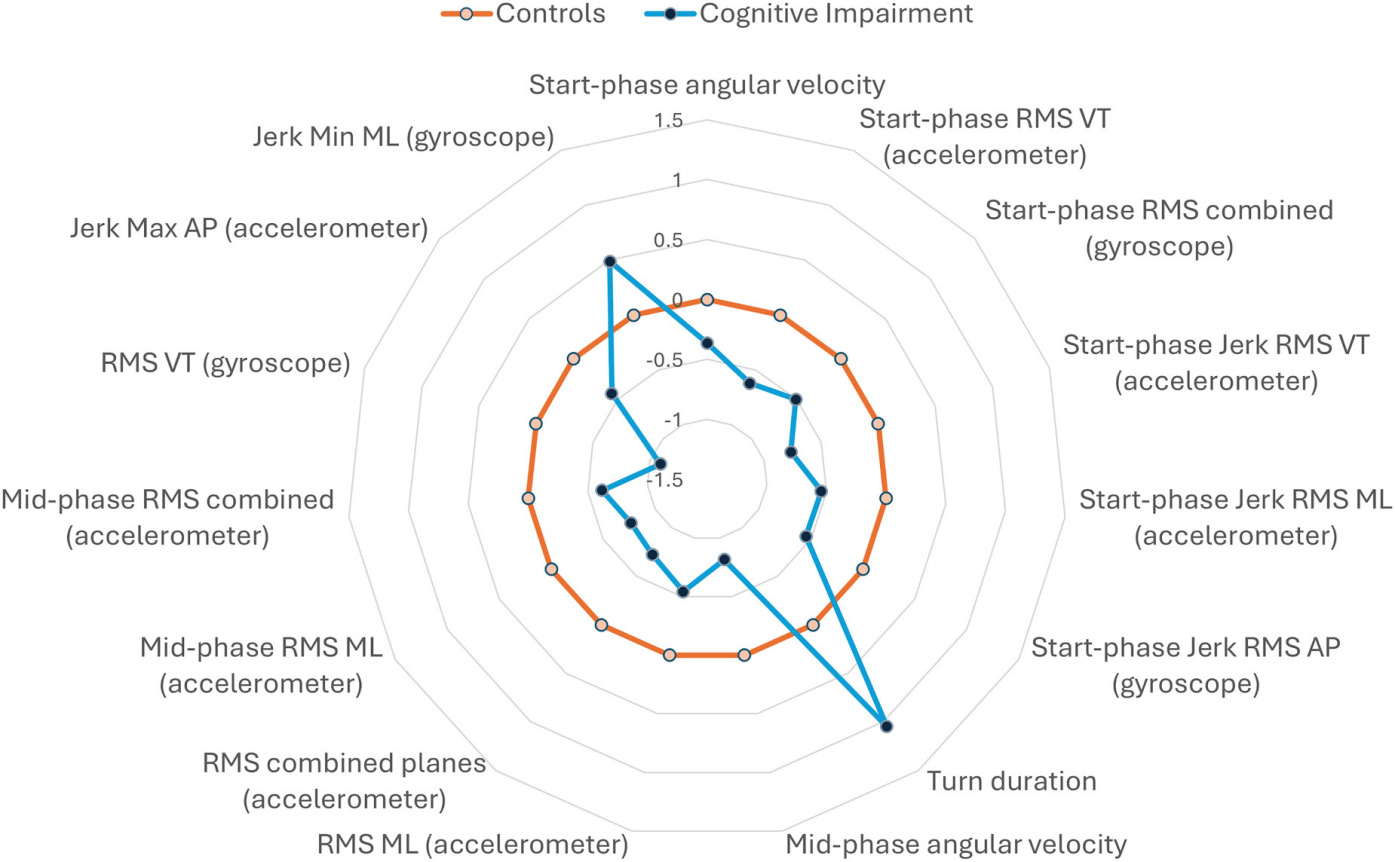
Radar plot illustrating differences in turning variables between cognitively impaired and control groups. The central orange line represents control data, and the blue line represents the cognitive impairment sample and demonstrates how many standard deviations from zero the group was (data were converted to z scores based on control means and standard deviations). RMS: root mean square; VT: vertical; ML: mediolateral; AP: anterior-posterior.

Following Bonferroni correction, the cognitively-impaired group showed significant differences compared to the control group, whereby they demonstrated lower values for variables relating to the Initiation domain: start-phase RMS in the vertical direction, start-phase jerk RMS in the vertical, mediolateral and anterior-posterior directions; and the Magnitude domain: mid-phase RMS in the mediolateral plane and mean RMS in the vertical planes. All significant variables demonstrated a moderate-strong effect size (see Table 2 for p-values and effect sizes, Figure 2 for illustration of group differences). Factor scores did not show significant differences between groups. A sensitivity analysis revealed no significant differences for any variable between MCI and dementia sub-groups following Bonferroni correction (see Supplemental Table 4 for group scores and Supplemental Figure 3 for boxplots depicting data distribution). A further sensitivity analysis, excluding people with Parkinson's disease cognitive impairment from the cognitively-impaired group, revealed similar findings; however, start phase jerk RMS in the anteroposterior direction (p = 0.006) and mean RMS in the vertical direction (p = 0.005) were no longer significant after Bonferroni correction was applied (see Supplemental Table 5).

**Table 2.**
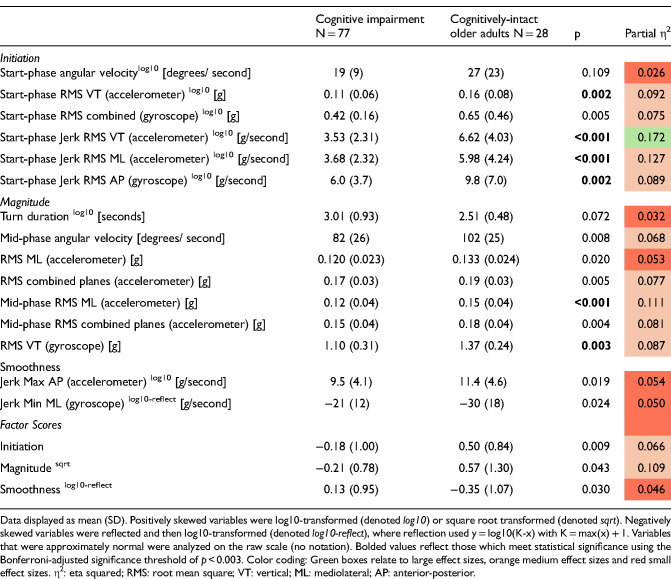
Between group comparisons for turning variables in cognitively impaired and control groups.

### Between-group differences and cognitive correlates with factor scores

**Figure 3.**
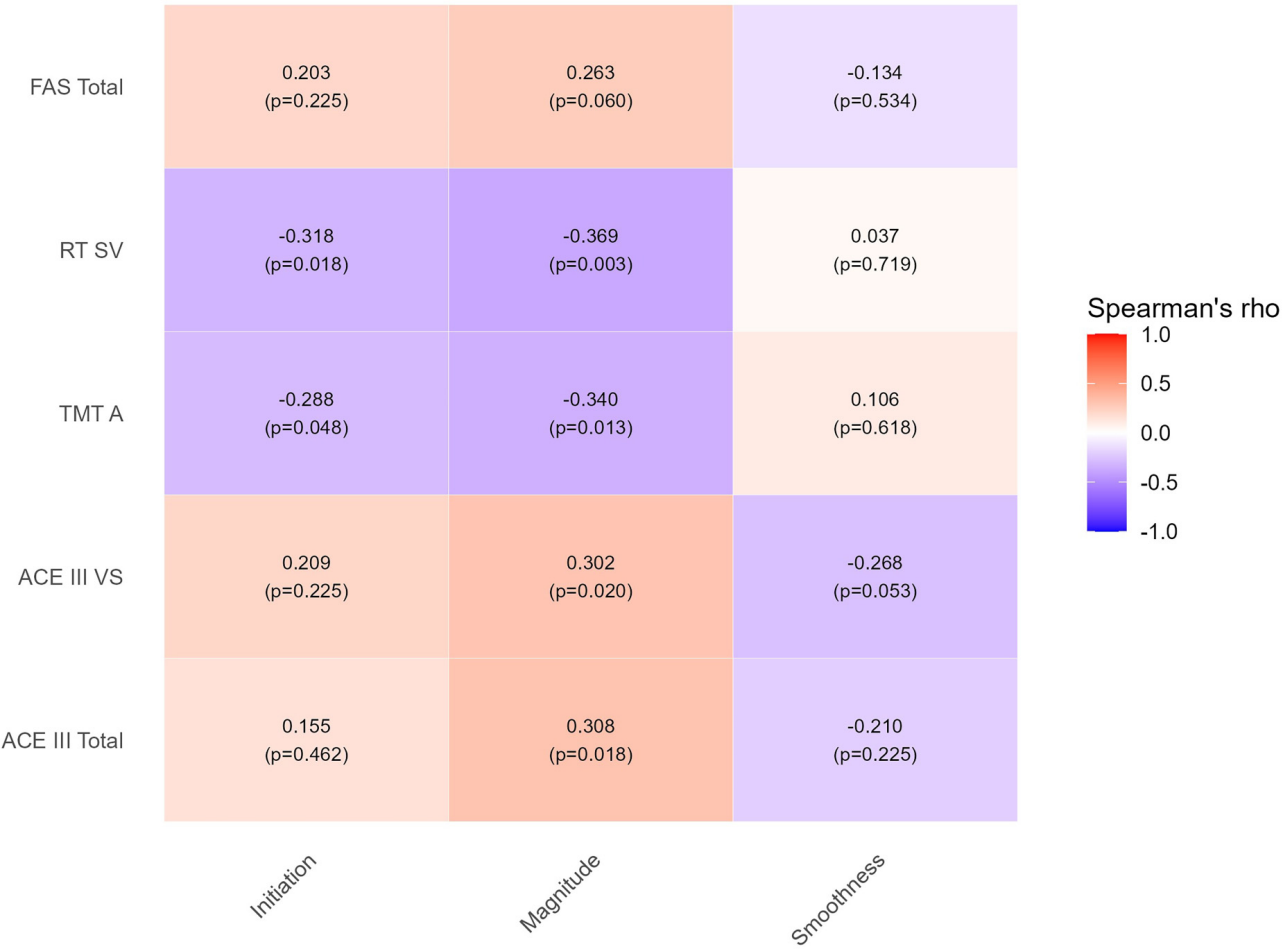
Heatmap of correlation coefficients (Spearman's Rho) between cognitive variables and turning factors.

Lower scores for both Turn Initiation and Magnitude were associated with longer attentional reaction time and information processing speeds, while lower scores for Magnitude were also associated with worse global cognition and visuospatial abilities (see Figure 3 for Spearman's rho and p values). Smoothness was not associated with any of the cognitive variables.

## Discussion

This study developed a novel data-driven model for turning performance relevant to people with MCI and dementia. It also comprehensively described turning performance in people with cognitive impairment compared to cognitively-intact older adults. Key results suggest three discrete turning domains: turn initiation, turn magnitude and turn smoothness. People with cognitive impairment demonstrated slower, more stable (lower RMS) movements during turn initiation with lower magnitude in both initiation and mid-phase of turns compared to cognitively-intact older adults, with moderate-large effect sizes; extending previous findings in MCI.^15^ Further research should consider a confirmatory factor analysis on a larger sample of individuals with cognitive impairment, and the potential use of these turning variables for predicting cognitive impairment in at-risk populations.

The derivation of different turning phases within this study also provides greater granularity to our understanding of turning performance in cognitive impairment. For example, smaller, stable movements during turn initiation may be related to slower turns as well as reduced postural adjustments (i.e., postural muscles recruited before a threat to balance occurs based on cognitive and sensory feedback to optimize control of the motor activity), as observed during gait initiation in neurological populations such as Parkinson's disease .^41,42^ Initiation of gait is considered a challenging task for the balance control system as it involves moving from a stable static state to a continuously unstable gait; likewise, turning is associated with instability and likely requires anticipatory physiological and cognitive processes to safely engage in this movement.^43,44^

Results suggest that both turn initiation and magnitude are associated with complex cognitive functions relating to anticipation, planning and execution (e.g., attention, information processing). These slower, more stable movements may reflect a cautious or compensatory approach to maintaining stability when cognitive resources are limited.^13,14^ This compensatory approach may have knock-on effects to the remainder of the turn maneuver, resulting in a lower magnitude and slowness of movement. This is evidenced further by velocity-related turn variables featuring in the initiation and mid-phase domains. However, there is a paucity of research investigating movement initiation during gait and turning in the cognitively impaired population, limiting our understanding of this potential mechanism. Associations with attention and information processing align with the limited studies investigating neural correlates of turning in Parkinson's disease, which suggest a potential mechanistic role of the prefrontal cortex,a neural region associated with higher-order cognitive functions involved in attention and information processing.^45,46^ However, significant gaps in our knowledge regarding the neural correlates of turning remain, particularly in people with cognitive impairment; therefore, we can only speculate on the underlying neural mechanisms that may drive turning impairments in this population. Future research should include people with cognitive impairment in neuroimaging studies pertaining to the neural mechanisms of turning to gain further insight.

We also note that turning factor scores did not show significant differences between groups after correcting for multiple comparisons and demonstrated smaller effect sizes than individual turning variables. This may suggest that summarizing across diverse turning variables reduces sensitivity to specific cognitive–motor alterations, or that the study was underpowered to detect small-to-moderate effects once conservative correction was applied. We note that this study included participants with Parkinson's disease cognitive impairment, a motor disorder which is known to be associated with turning impairments. It is possible the inclusion of this dementia subtype may have influenced the results, however a sensitivity analysis removing this group did not significantly alter the interpretation of our findings. We can therefore postulate that the differences found in turning performance between the cognitive impairment group and cognitively-intact older adults were unlikely to be driven by Parkinsonism motor features and may reflect changes associated with cognitive impairment more broadly.

Although some turning variables differed between MCI and dementia sub-groups at the uncorrected level, these differences did not remain significant after Bonferroni correction. This finding was unsurprising as the same cohort showed no differences between MCI and dementia for gait impairments,^11^ and the wider literature, which has focused primarily on Alzheimer's disease rather than all-cause dementia, reports inconsistent gait differences between these groups.^9^ Our findings may suggest that turning requires compensatory strategies early in the course of cognitive impairment (i.e., slowing to maintain stability). This would reflect the wealth of evidence which reports gait impairments years before the onset of apparent cognitive symptoms; however, inferences are limited by the cross-sectional nature of these data.^2–4^ Future research should consider the potential for turning variables, particularly those relating to turn initiation, in predicting incidence of cognitive impairment in at-risk groups (e.g., rapid eye movement (REM) sleep behavior disorder, Parkinson's disease) to understand clinical relevance. This would also allow further validation of the proposed conceptual model's robustness. In line with the gait literature, turning performance may differ between more severe stages of dementia compared to the mild stage group included in this study; however, this was not possible with our current sample. We therefore recommend future research considers assessing turning performance longitudinally to understand how it changes with further cognitive decline, and how this might support markers of disease or clinical endpoints.^47^ Additionally, comparison of turning performance between dementia subtypes might be considered in the future, as complementary additions to emerging evidence of gait as a supportive differential marker.^11,12^

This study has several strengths. A well-characterized sample of cognitively impaired participants were included, with clinical consensus adding confidence to diagnoses associated with dementia. The algorithm to derive turning measures has previously been validated in this sample, and is considered appropriate for use across the common dementia subtypes included in the present analyses.^25^ Data reduction was guided by literature on cognitive correlates of turning in neurological conditions, and reduction of 102 variables to 15 across three domains improves interpretability and clinical translatability of results.^13–15^ However, this may have restricted identification of more complex patterns in the data. Future research could consider employing machine-learning feature reduction techniques to complement this approach and explore the predictive value of turning in prodromal cohorts, its sensitivity to disease changes over time and responsiveness to intervention.

This study also had some limitations. Although the sample size is comparable with previous efforts in gait amongst neurological conditions,^27^ the sample size is relatively low for conducting a factor analysis (105 participants: 15 variables, participants-per-variable: 7:1). Although there are no standardized guidelines, usual recommendations suggest 200–1000 participants, or participants-per-variable range from 4–1 and 20–1.^38^ Future research should consider conducting confirmatory factor analysis in a larger cohort. Turning variables were collected in controlled conditions whereby participants were instructed to undertake a clinical walking assessment involving 180° turns; this protocol likely requires a level of anticipatory turning, reflected by the turning initiation domain, and may not replicate during more continuous turns (as found during continuous circuit walking assessments) or the in the real world, both of which may require different strategies for planning and execution. Finally, although our control participants were age-matched and recruited from the same local area, they demonstrated a higher premorbid IQ and fewer co-morbidities than those with cognitive impairment; this may suggest that they represent a relatively healthy and high-functioning sample. While this is not surprising, given that people with multi-morbidity and lower education or IQ are more likely to develop cognitive impairment,^48,49^ findings of this study should be interpreted in light of this and future research should make targeted efforts to recruit a more inclusive control cohort to replicate findings.^50,51^

In conclusion, this novel exploratory study developed a framework to measure turning performance in people with cognitive impairment using inertial measurement units. Variables relating to turn initiation appeared most prominently affected by cognitive impairment and may reflect compensatory physiological processes to offset limited cognitive resource. Further research should consider the use of this framework in predicting onset of cognitive impairment in at-risk populations, and to consider its use in supporting differential dementia subtype diagnosis.

## Supplemental Material

sj-docx-1-alz-10.1177_13872877261450969 - Supplemental material for Laboratory-based turning performance during walking in people with mild cognitive impairment and dementia Supplemental material, sj-docx-1-alz-10.1177_13872877261450969 for Laboratory-based turning performance during walking in people with mild cognitive impairment and dementia by Ríona Mc Ardle, Andrew Kingston, Silvia Del Din, Rana Zia Ur Rehman, Brook Galna, Alan J. Thomas, Lynn Rochester and Lisa Alcock in Journal of Alzheimer's Disease

## References

[1] Prince M Wimo A Guerchet M , et al. World Alzheimer report 2015. The global impact of dementia: an analysis of prevalence, incidence, cost and trends. London: Alzheimer's Disease International, 2015.

[2] Beauchet O Annweiler C Callisaya ML , et al. Poor gait performance and prediction of dementia: results from a meta-analysis. J Am Med Dir Assoc 2016; 17: 482–490. 26852960 10.1016/j.jamda.2015.12.092 PMC5319598

[3] Buracchio T Dodge HH Howieson D , et al. The trajectory of gait speed preceding mild cognitive impairment. Arch Neurol 2010; 67: 980–986. 20697049 10.1001/archneurol.2010.159 PMC2921227

[4] Blumen HM Jayakody O Verghese J . Gait in cerebral small vessel disease, pre-dementia, and dementia: a systematic review. Int J Stroke 2023; 18: 53–61. 35797006 10.1177/17474930221114562 PMC9841467

[5] Mc Ardle R Morris R Hickey A , et al. Gait in mild Alzheimer's disease: feasibility of multi-center measurement in the clinic and home with body-worn sensors: a pilot study. J Alzheimers Dis 2018; 63: 331–341. 29614664 10.3233/JAD-171116 PMC7617011

[6] Verghese J . Gait and cognitive declines in dementia-double or nothing. JAMA Netw Open 2022; 5: e2214654. 10.1001/jamanetworkopen.2022.14654 35639385

[7] Montero-Odasso M Speechley M Muir-Hunter SW , et al. Motor and cognitive trajectories before dementia: results from gait and brain study. J Am Geriatr Soc 2018; 66: 1676–1683. 29608780 10.1111/jgs.15341

[8] Montero-Odasso M Speechley M Muir-Hunter SW , et al. Dual decline in gait speed and cognition is associated with future dementia: evidence for a phenotype. Age Ageing 2020; 49: 995–1002. 32559288 10.1093/ageing/afaa106 PMC7583522

[9] Mc Ardle R Morris R Wilson J , et al. What can quantitative gait analysis tell us about dementia and its subtypes? A structured review. J Alzheimers Dis 2017; 60: 1295–1312. 29036826 10.3233/JAD-170541

[10] Mc Ardle R Del Din S Galna B , et al. Differentiating dementia disease subtypes with gait analysis: feasibility of wearable sensors? Gait Posture 2020; 76: 372–376. 31901765 10.1016/j.gaitpost.2019.12.028

[11] Mc Ardle R Galna B Donaghy P , et al. Do Alzheimer's and Lewy body disease have discrete pathological signatures of gait? Alzheimers Dement 2019; 15: 1367–1377. 31548122 10.1016/j.jalz.2019.06.4953

[12] Elasfar S Hameed H Boeve BF , et al. Identifying gait differences between Alzheimer's disease and dementia with Lewy bodies and their associations with regional amyloid deposition. Alzheimers Dement 2025; 21: e14351. 10.1002/alz.14351 PMC11815204 39868511

[13] Mancini M Schlueter H El-Gohary M , et al. Continuous monitoring of turning mobility and its association to falls and cognitive function: a pilot study. J Gerontol A Biol Sci Med Sci 2016; 71: 1102–1108. 26916339 10.1093/gerona/glw019 PMC5007616

[14] Morris R Martini DN Smulders K , et al. Cognitive associations with comprehensive gait and static balance measures in Parkinson's disease. Parkinsonism Relat Disord 2019; 69: 104–110. 31731260 10.1016/j.parkreldis.2019.06.014 PMC6900452

[15] Mirelman A Weiss A Buchman AS , et al. Association between performance on timed up and go subtasks and mild cognitive impairment: further insights into the links between cognitive and motor function. J Am Geriatr Soc 2014; 62: 673–678. 24635699 10.1111/jgs.12734 PMC3989433

[16] Morris R Lord S Bunce J , et al. Gait and cognition: mapping the global and discrete relationships in ageing and neurodegenerative disease. Neurosci Biobehav Rev 2016; 64: 326–345. 26915926 10.1016/j.neubiorev.2016.02.012

[17] Maidan I Bernad-Elazari H Giladi N , et al. When is higher level cognitive control needed for locomotor tasks among patients with Parkinson’s disease? Brain Topogr 2017; 30: 531–538. 28439757 10.1007/s10548-017-0564-0

[18] Leach JM Mellone S Palumbo P , et al. Natural turn measures predict recurrent falls in community-dwelling older adults: a longitudinal cohort study. Sci Rep 2018; 8: 4316. 29531284 10.1038/s41598-018-22492-6 PMC5847590

[19] Gulley E Ayers E Verghese J . A comparison of turn and straight walking phases as predictors of incident falls. Gait Posture 2020; 79: 239–243. 32450510 10.1016/j.gaitpost.2020.05.002 PMC7299744

[20] Kamil RJ Bakar D Ehrenburg M , et al. Detection of wandering behaviors using a body-worn inertial sensor in patients with cognitive impairment: a feasibility study. Front Neurol 2021; 12: 529661. 33776875 10.3389/fneur.2021.529661 PMC7991404

[21] Schmidt L Zieschang T Koschate J , et al. Impaired standing balance in older adults with cognitive impairment after a severe fall. Gerontology 2024; 70: 755–763. 38679005 10.1159/000538598

[22] Mancini M El-Gohary M Pearson S , et al. Continuous monitoring of turning in Parkinson's disease: rehabilitation potential. Neurorehabilitation 2015; 37: 3–10. 26409689 10.3233/NRE-151236 PMC4745985

[23] Weston AR Lohse KR Kittelson A , et al. Turning speed as a more responsive metric of age-related decline in mobility: a comparative study with gait speed. Clin Biomech (Bristol) 2024; 113: 106196. 38354515 10.1016/j.clinbiomech.2024.106196 PMC10955671

[24] Rehman RZU Klocke P Hryniv S , et al. Turning detection during gait: algorithm validation and influence of sensor location and turning characteristics in the classification of Parkinson's disease. Sensors (Basel) 2020; 20: 5377. 32961799 10.3390/s20185377 PMC7570702

[25] Mc Ardle R Ryan LJ Rehman RZU , et al. Validation of an algorithm for detecting turning in people with cognitive impairment, considering dementia disease subtype. Gait Posture 2025; 118: 141–147. 39970572 10.1016/j.gaitpost.2025.02.011

[26] Arcolin I Corna S Giardini M , et al. Proposal of a new conceptual gait model for patients with Parkinson's disease based on factor analysis. Biomed Eng Online 2019; 18: 70. 31159825 10.1186/s12938-019-0689-3 PMC6547597

[27] Monaghan AS Huisinga JM Peterson DS . The application of principal component analysis to characterize gait and its association with falls in multiple sclerosis. Sci Rep 2021; 11: 12811. 34140612 10.1038/s41598-021-92353-2 PMC8211858

[28] McKhann GM Knopman DS Chertkow H , et al. The diagnosis of dementia due to Alzheimer’s disease: recommendations from the national institute on aging-Alzheimer’s association workgroups on diagnostic guidelines for Alzheimer's disease. Alzheimers Dement 2011; 7: 263–269. 21514250 10.1016/j.jalz.2011.03.005 PMC3312024

[29] McKeith IG Boeve BF Dickson DW , et al. Diagnosis and management of dementia with Lewy bodies: fourth consensus report of the DLB consortium. Neurology 2017; 89: 88–100. 28592453 10.1212/WNL.0000000000004058 PMC5496518

[30] McKeith IG Ferman TJ Thomas AJ , et al. Research criteria for the diagnosis of prodromal dementia with Lewy bodies. Neurology 2020; 94: 743–755. 32241955 10.1212/WNL.0000000000009323 PMC7274845

[31] Emre M Aarsland D Brown R , et al. Clinical diagnostic criteria for dementia associated with Parkinson's disease. Mov Disord 2007; 22: 1689–1707; quiz 1837. 17542011 10.1002/mds.21507

[32] Litvan I Goldman JG Troster AI , et al. Diagnostic criteria for mild cognitive impairment in Parkinson's disease: movement disorder society task force guidelines. Mov Disord 2012; 27: 349–356. 22275317 10.1002/mds.24893 PMC3641655

[33] Albert MS DeKosky ST Dickson D , et al. The diagnosis of mild cognitive impairment due to Alzheimer's disease: recommendations from the national institute on aging-Alzheimer's association workgroups on diagnostic guidelines for Alzheimer's disease. Alzheimers Dement 2011; 7: 270–279. 21514249 10.1016/j.jalz.2011.03.008 PMC3312027

[34] Roman GC Tatemichi TK Erkinjuntti T , et al. Vascular dementia: diagnostic criteria for research studies. Report of the NINDS-AIREN international workshop. Neurology 1993; 43: 250–260. 8094895 10.1212/wnl.43.2.250

[35] Goetz CG Tilley BC Shaftman SR , et al. Movement disorder society-sponsored revision of the unified Parkinson's disease rating scale (MDS-UPDRS): scale presentation and clinimetric testing results. Mov Disord 2008; 23: 2129–2170. 19025984 10.1002/mds.22340

[36] Ciafone J Thomas A Durcan R , et al. Neuropsychological impairments and their cognitive architecture in mild cognitive impairment (MCI) with Lewy bodies and MCI-Alzheimer's disease. J Int Neuropsychol Soc 2022; 28: 963–973. 34666864 10.1017/S1355617721001181

[37] Cohen J . Statistical power analysis for the behavioral sciences. New York: Routledge, 2013.

[38] Williams B Onsman A Brown T . Exploratory factor analysis: a five-step guide for novices. Australasian J Paramed 2010; 8: 1–13.

[39] Norman GR Streiner DL . Biostatistics: the bare essentials. Shelton, CT: People's Medical Publishing House, 2008.

[40] Kaiser HF . The application of electronic computers to factor analysis. Educ Psychol Meas 1960; 20: 141–151.

[41] Mancini M Zampieri C Carlson-Kuhta P , et al. Anticipatory postural adjustments prior to step initiation are hypometric in untreated Parkinson's disease: an accelerometer-based approach. Eur J Neurol 2009; 16: 1028–1034. 19473350 10.1111/j.1468-1331.2009.02641.x PMC2840629

[42] Halliday SE Winter DA Frank JS , et al. The initiation of gait in young, elderly, and Parkinson's disease subjects. Gait Posture 1998; 8: 8–14. 10200394 10.1016/s0966-6362(98)00020-4

[43] Bayot M Dujardin K Tard C , et al. The interaction between cognition and motor control: a theoretical framework for dual-task interference effects on posture, gait initiation, gait and turning. Neurophysiol Clin 2018; 48: 361–375. 30487064 10.1016/j.neucli.2018.10.003

[44] Russo Y Leveridge P Ye J , et al. Characterising anticipatory postural adjustments in turning a comparison between older adults and people with Parkinson's disease. Sci Rep 2025; 15: 45125. 41429909 10.1038/s41598-025-33425-5 PMC12748813

[45] Belluscio V Stuart S Bergamini E , et al. The association between prefrontal cortex activity and turning behavior in people with and without freezing of gait. Neuroscience 2019; 416: 168–176. 31330231 10.1016/j.neuroscience.2019.07.024 PMC7778469

[46] Stuart S Belluscio V Quinn JF , et al. Pre-frontal cortical activity during walking and turning is reliable and differentiates across young, older adults and people with Parkinson's disease. Front Neurol 2019; 10: 536. 31191434 10.3389/fneur.2019.00536 PMC6540937

[47] Elshehabi M Hansen C Hobert MA , et al. Turning slowly predicts future diagnosis of Parkinson's disease: a decade-long longitudinal analysis. Ann Neurol 2026; 99: 114–123. 41117503 10.1002/ana.78034 PMC12946604

[48] Shepherd H Todd A Sinclair DR , et al. The association between multiple long-term conditions and dementia: a UK cohort study. Alzheimers Dement (Amst) 2025; 17: e70230. 10.1002/dad2.70230 PMC12703650 41404483

[49] Livingston G Huntley J Liu KY , et al. Dementia prevention, intervention, and care: 2024 report of the lancet standing commission. Lancet 2024; 404: 572–628. 39096926 10.1016/S0140-6736(24)01296-0

[50] Goodwin VA Low MSA Quinn TJ , et al. Including older people in health and social care research: best practice recommendations based on the INCLUDE framework. Age Ageing 2023; 52: afad082. 10.1093/ageing/afad082 PMC10234283 37261448

[51] Witham MD Anderson E Carroll CB , et al. Ensuring that COVID-19 research is inclusive: guidance from the NIHR INCLUDE project. BMJ Open 2020; 10: e043634. 10.1136/bmjopen-2020-043634 PMC7646322 33154065

