# Supplementary material for: Laboratory-based turning performance during walking in people with mild cognitive impairment and dementia
Source: J Alzheimers Dis. 2026 May 27;112(1):302–12. doi: 10.1177/13872877261450969 (PMC13291383; doi:10.1177/13872877261450969)
Supplement: sj-docx-1-alz-10.1177_13872877261450969 - Supplemental material for Laboratory-based turning performance during walking in people with mild cognitive impairment and dementia [file sj-docx-1-alz-10.1177_13872877261450969.docx]

**Supplemental Material**

**Laboratory-based turning performance during walking in people with mild cognitive impairment and dementia**

**Full details of methods and results pertaining to the factor analysis for turning performance**

*Statistical analysis pertaining to the factor analysis*

Statistical analysis pertaining to the factor analysis was divided into two phases: (1) Data reduction: (a) Association between turning variables with key cognitive domains; (b) Correlation matrix between turning variables; and (2) Exploratory factor analysis (EFA), essential for creating a novel model of turning in our sample of people with cognitive impairment.

*1a. Association between turning variables and key cognitive domains.*

In order to reduce the number of turning variables and identify candidate variables for the EFA, associations between cognitive scores relating to global cognition, attention, information processing, executive function and visuospatial ability with turning variables were examined using Spearman’s rank correlations in the whole dataset (including cognitively-intact older adults). Given the exploratory nature of this analysis, turning variables were retained for the subsequent correlation matrix if they showed a correlation coefficients ≥ 0.30 for at least one correlation with a cognitive measure.[^1^](#_ENREF_1) Formal significance testing was not considered to avoid loss of potentially relevant variables.

*1b. Correlation matrix*

The correlation matrix used for factor analysis was derived solely from the cognitive impairment group. This was to capture relationships between turning variables that are clinically meaningful in this group. For EFA, only the variables with Spearman’s rank correlation coefficients ≥ 0.30 but < 0.90 were considered.[^1^](#_ENREF_1)^,^ [^2^](#_ENREF_2) The Kaiser-Meyer-Olkin (KMO) sampling adequacy was run: KMO values for each single variable of >0.50 and overall KMO of >0.70 were considered acceptable for analysis.[^3^](#_ENREF_3) The Bartlett’s test of Sphericity was checked for significance to ensure a factor analysis was suitable.[^4^](#_ENREF_4)

*2. Exploratory factor analysis*

EFA was conducted using principal components as the extraction method with the variables selected from the correlation matrix. EFA is used to determine if numerous measures can be explained by a smaller number of factors.[^2^](#_ENREF_2)^,^ [^3^](#_ENREF_3) The following criteria was used to identify the number of factors: (1) Including only factors with an eigenvalue >1, as per Kaiser’s criteria;[^5^](#_ENREF_5) (2) Cattell’s Scree Test which allows visual examination of a plot of eigenvalues for each factor and allows identification of the breakpoint of the scree – factors before this breakpoint are retained;[^5^](#_ENREF_5) (3) Horn’s parallel analysis, which involves generation of a synthetic dataset which is superimposed on the Scree plot of real data, with only factors whose eigen value is greater than that of the random data are retained.[^6^](#_ENREF_6)

Once the number of factors were determined, factor rotation was conducted to assess the distribution of variables onto factors. As factors were expected to be correlated, a Promax oblique rotation method was used.[^2^](#_ENREF_2) This produced correlation coefficients of variables, which were then evaluated for retention using statistical and conceptual criteria. Primary loadings were retained if the value was ≥ 0.50 and the difference between the highest and second-highest absolute loadings was ≥ 0.20, to minimize effects of cross-loading.[^2^](#_ENREF_2)

Given the inclusion of participants with Parkinson’s disease cognitive impairment, who may exhibit motor-related turning impairments, a sensitivity analysis was performed by excluding these participants (n = 10) from the sample and re-running the exploratory factor analysis.

**Results**

*Associations between cognitive domains and turning variables*

Associations between five cognitive scores and 95 turning variables were initially considered, with 33 turning variables showing a Spearman Rho correlation ≥ 0.3 with at least one cognitive score; these were retained for subsequent analysis. Supplemental Table 1 shows a heatmap of correlation coefficients between cognitive scores and retained turning variables, while Supplemental Table 2 reports excluded variables at this phase.

*Correlation matrix*

Based on the correlation matrix, a further ten turning variables were excluded due to redundancy (absolute correlation >0.90 with another variable; see Supplemental Figure 1). After this selection process, 23 variables remained for the factor analysis. The overall KMO measure was 0.86, suggesting adequacy for producing distinct factors with EFA.

*Exploratory factor analysis*

Based on *a priori* criteria, four factors were determined. An initial EFA was conducted on the 23 turning variables, and the rotated factor pattern was inspected for cross-loadings. Eight variables failing to meet a-priori criterion regarding factor loadings were removed to improve structure and interpretability. Following removal, l5 turning variables were retained with an overall KMO measure of 0.82 (Supplemental Figure 2), and the EFA was re-run. Three factors were revealed using previously described criteria., which accounted for 67% of the total variance (Turn Initiation: 27%, Turn Magnitude: 30%, Turn Smoothness: 10%). Factor loadings are reported in Figure 1. Our sensitivity analysis, whereby we removed participants with Parkinson’s disease cognitive impairment, revealed a highly consistent structure compared to the primary analysis reported here (see Supplemental Table 3).The same turning domains were yielded and accounted for 67% of the variance (Turn Initiation: 28%, Turn Magnitude: 29%, Turn Smoothness: 10%). Two variables (start phase RMS in the vertical direction and mean RMS in combined planes) did not meet the loading criteria (<0.50); however, this did not change interpretation.

**References**

1. Cohen J. *Statistical power analysis for the behavioral sciences*. New York: Rutledge, 2013.

2. Williams B, Onsman A and Brown T. Exploratory factor analysis: A five-step guide for novices. *Australasian J Paramed* 2010; 8: 1-13.

3. Norman GR and Streiner DL. *Biostatistics: the bare essentials*. PMPH USA (BC Decker), 2008.

4. Tobias S and Carlson JE. Brief Report: Bartlett's test of sphericity and chance findings in factor analysis. *Multivariate Behav Res* 1969; 4: 375-377.

5. Kaiser HF. The application of electronic computers to factor analysis. *Educ Psychol Measure* 1960; 20: 141-151.

6. Horn JL. A rationale and test for the number of factors in factor analysis. *Psychometrika* 1965; 30: 179-185.

**Supplemental Table 1.** Demographic and clinical information for participants, split by cognitive status.

|  | **N** | **Mild Cognitive Impairment**  N = 31 | **Dementia**  N=46 | **Cognitively Intact Older Adults**  N = 28 |
| --- | --- | --- | --- | --- |
| Subtype | 105 |  |  |  |
| Alzheimer’s disease |  | 15 (48%) | 20 (43%) |  |
| Dementia with Lewy Bodies |  | 10 (32%) | 15 (33%) |  |
| Parkinson’s disease cognitive impairment |  | 4 (13%) | 6 (13%) |  |
| Vascular dementia |  | 2 (6.5%) | 5 (11%) |  |
| Age (years) | 105 | 76 (65-91) | 76 (66-87) | 73 (60-89) |
| Sex (male) | 105 | 18 (58%) | 32 (70%) | 11 (39%) |
| Height (m) | 105 | 1.69 (1.45-1.81) | 1.70 (1.51-1.87) | 1.67 (1.52-1.83) |
| UPDRS III (n/132) | 101 | 16 (0-70) | 14 (0-69) | 1 (0-11) |
| sMMSE (n/30) | 105 | 26.0 (21.0-30.0) | 22.0 (14.0-28.0) | 30 (25-30) |
| ACE-III Total (n/100) | 104 | 82 (64-95) | 69 (15-88) | 97 (87-100) |
| ACE-III VS (n/16) | 104 | 15 (9-16) | 13 (0-16) | 16 (13-16) |
| FAS Total | 102 | 37 (7-61) | 27 (10-59) | 45 (29-69) |
| TMT A (secs) | 94 | 53 (24-720) | 69 (28-955) | 30 (19-65) |
| Simple RT (secs) | 99 | 409 (287-748) | 472 (287-3,792) | 372 (291-493) |
| CIRSG (n/56) | 104 | 8 (3-18) | 10 (4-19) | 4 (0-11) |
| NART | 103 | 118 (101-125) | 116 (100-125) | 123 (114-126) |
| Parkinson’s medication (% Yes) | 105 | 4 (13%) | 9 (20%) | 0 (0%) |
| Dementia treatment (% Yes) | 98 | 16 (59%) | 32 (74%) | 0 (0%) |

N(%), median (range); MCI: mild cognitive impairment; UPDRS-III: Movement Disorders Society Unified Parkinson’s disease rating scale; sMMSE: standardized Mini-Mental State Examination; ACE-III: Addenbrooke’s Cognitive Examination; VS: visuospatial; FAS: FAS verbal fluency test; TMT A: Trail making test A; RT: reaction time; CIRSG: Cumulative illness rating scale – geriatric; NART: National Adult Reading Test (IQ)

**Supplemental Table 2.** Demographic and clinical information for participants, split by dementia subtype.

|  | **N** | **Alzheimer’s disease**  N = 35 | **Dementia with Lewy bodies**  N=25 | **Parkinson’s disease cognitive impairment**  **N=10** | **Vascular dementia**  **N=7** | **Cognitively Intact Older Adults** N = 28 |
| --- | --- | --- | --- | --- | --- | --- |
| Disease severity | 105 |  |  |  |  |  |
| MCI |  | 15 (43%) | 10 (40%) | 4 (40%) | 2 (29%) | n/a |
| Dementia |  | 20 (57%) | 15 (60%) | 6 (60%) | 5 (71%) | n/a |
| Age (years) | 105 | 76 (67-88) | 75 (65-89) | 77 (70-91) | 83 (66-85) | 73 (60-89) |
| Sex (male) | 105 | 14 (40%) | 21 (84%) | 9 (90%) | 6 (86%) | 11 (39%) |
| Height (m) | 105 | 1.65 (1.45-1.86) | 1.71 (1.57-1.87) | 1.66 (1.57-1.79) | 1.77 (1.48-1.83) | 1.67 (1.52-1.83) |
| UPDRS III (n/132) | 101 | 7 (0-19) | 26 (5-57) | 41 (0-70) | 23 (5-30) | 1 (0-11) |
| sMMSE (n/30) | 105 | 23 (14-29) | 25 (16-30) | 24 (21-30) | 23 (15-28) | 30 (25-30) |
| ACE-III Total (n/100) | 104 | 74 (28-90) | 77 (15-95) | 81 (63-95) | 72 (41-86) | 97 (87-100) |
| ACE-III VS (n/16) | 104 | 14 (6-16) | 12 (0-16) | 1 (11-16) | 14 (7-16) | 16 (13-16) |
| FAS Total | 102 | 36 (11-61) | 30 (7-58) | 21 (11-48) | 18 (10-35) | 45 (29-69) |
| TMT A (secs) | 94 | 49 (29-250) | 101 (28-835) | 119 (24-955) | 96 (57-125) | 30 (19-65) |
| Simple RT (secs) | 99 | 408 (287-773) | 430 (287-1,071) | 572 (386-3,792) | 605 (315-1,494) | 372 (291-493) |
| CIRSG (n/56) | 104 | 8 (3-19) | 10 (4-18) | 10 (3-17) | 12 (6-15) | 4 (0-11) |
| NART | 103 | 117 (101-125) | 116 (101-124) | 120 (113-123) | 118 (100-124) | 123 (114-126) |
| Parkinson’s medication (% Yes) | 105 | 0 (0%) | 3 (12%) | 10 (100%) | 0 (0%) | 0 (0%) |
| Dementia treatment (% Yes) | 98 | 19 (66%) | 19 (79%) | 8 (80%) | 2 (29%) | 0 (0%) |

N(%), median (range); MCI: mild cognitive impairment; UPDRS-III: Movement Disorders Society Unified Parkinson’s disease rating scale; sMMSE: standardized Mini-Mental State Examination; ACE-III: Addenbrooke’s Cognitive Examination; VS: visuospatial; FAS: FAS verbal fluency test; TMT A: Trail making test A; RT: reaction time; CIRSG: Cumulative illness rating scale – geriatric; NART: National Adult Reading Test (IQ)

**Supplemental Figure 1.** Heatmap of correlation coefficients (Spearman’s Rho) between cognitive and turning variables.


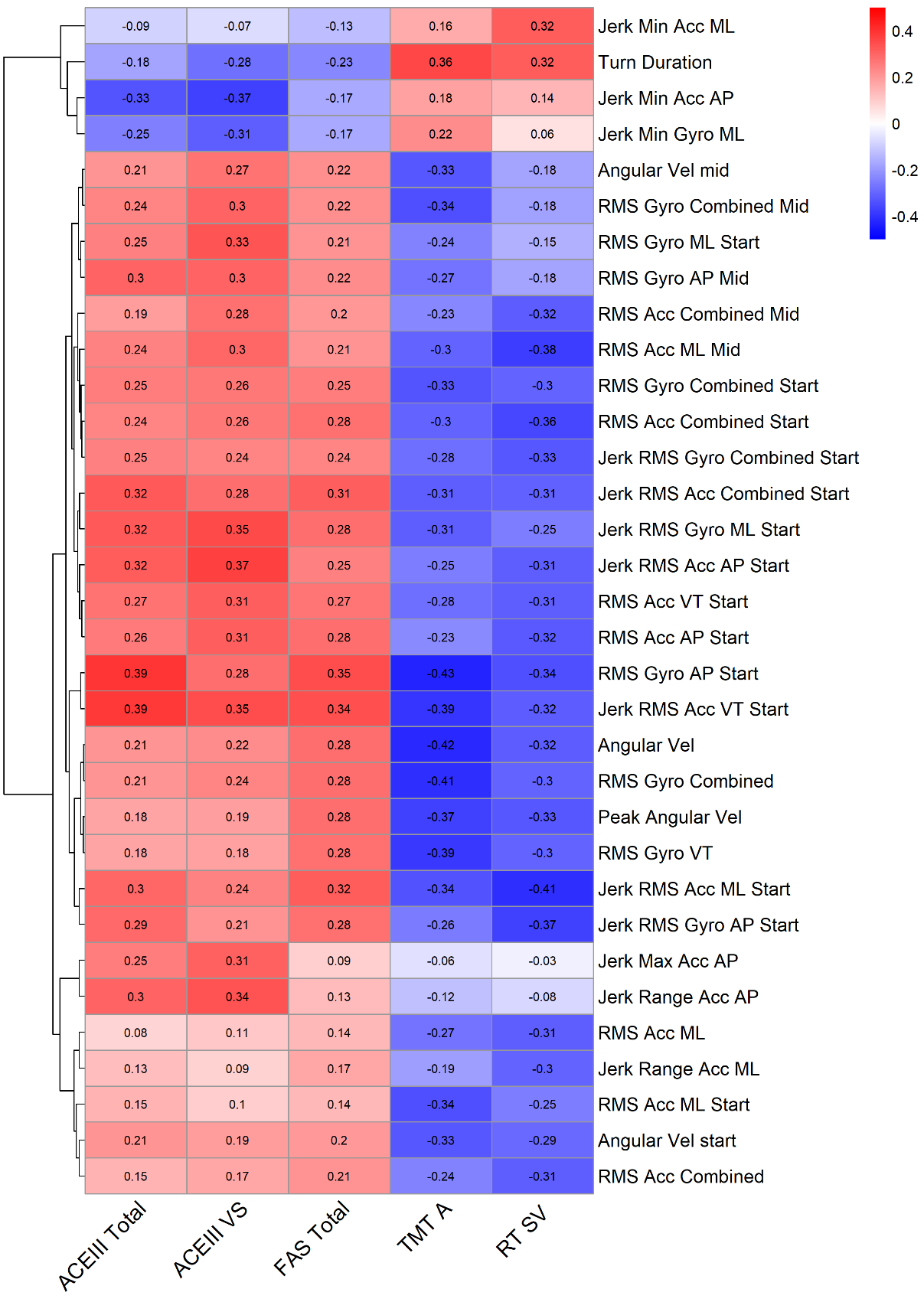


ACE-III: Addenbrooke’s Cognitive Examination; VS: visuospatial; FAS: FAS verbal fluency test; TMT A: Trail making test A; RT SV: reaction time single vigilance task; Acc: outcome derived from the accelerometer signal; gyro: outcome derived from the gyroscope signal; RMS: root mean square; VT: vertical; ML: mediolateral; AP: anterior-posterior; start: start-phase; mid: mid-phase; end: end-phase

**Supplemental Figure 2.** Flow chart describing data reduction and selection of final turning variables.


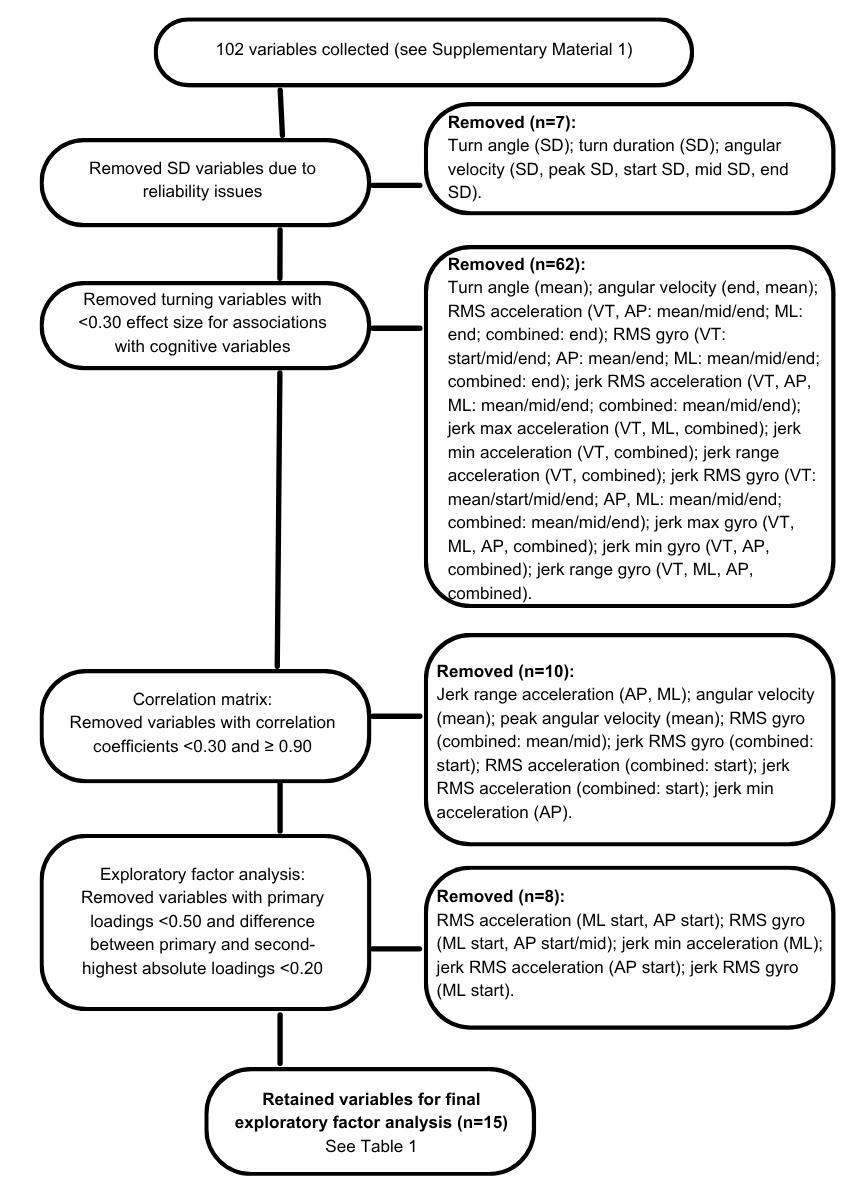


RMS: root mean square; VT: vertical axis; ML: mediolateral; AP: anterior-posterior

**Supplemental Table 3.** Factor loadings for turning performance when Parkinson’s disease cognitive impairment participants (n=10) are removed.

| **Variable** | **Initiation** | **Magnitude** | **Smoothness** |
| --- | --- | --- | --- |
| Start-phase Jerk RMS ML | 0.932 |  |  |
| Start-phase Jerk RMS VT | 0.871 |  |  |
| Start-phase Jerk RMS AP | 0.868 |  |  |
| Start-phase RMS combined | 0.824 |  |  |
| Start-phase angular velocity | 0.724 |  |  |
| RMS VT |  | 0.868 |  |
| Mid-phase RMS combined planes |  | 0.831 |  |
| Mid-phase angular velocity |  | 0.821 |  |
| Mid-phase RMS ML |  | 0.765 |  |
| RMS ML |  | 0.711 |  |
| Turn duration |  | -0.684 |  |
| Jerk Min ML |  |  | 0.839 |
| Jerk Max AP |  |  | -0.771 |

**Supplemental Table 4.** Between-group comparisons for turning variables for MCI and dementia subgroups.

|  | **Dementia**  N = 46 | **MCI**  N = 31 | **p** |
| --- | --- | --- | --- |
| **Initiation** |  |  |  |
| Start-phase angular velocity [degrees/second] | 18 (7) | 22 (11) | 0.043 |
| Start-phase RMS VT (accelerometer) ^log10^ [g] | 0.10 (0.04) | 0.13 (0.09) | 0.132 |
| Start-phase RMS combined (gyroscope) [g] | 0.39 (0.12) | 0.45 (0.19) | 0.154 |
| Start-phase Jerk RMS VT (accelerometer) ^log10^ [g/second] | 3.05 (1.52) | 4.25 (3.03) | 0.100 |
| Start-phase Jerk RMS ML (accelerometer) ^sqrt^ [g/second] | 3.29 (1.78) | 4.26 (2.88) | 0.069 |
| Start-phase Jerk RMS AP (gyroscope) ^log10^ [g/second] | 5.4 (3.5) | 6.9 (3.8) | 0.052 |
| **Magnitude** |  |  |  |
| Turn duration ^log10^ [seconds] | 3.06 (1.05) | 2.93 (0.74) | 0.822 |
| Mid-phase angular velocity [degrees/second] | 82 (25) | 82 (27) | 0.935 |
| RMS ML (accelerometer) [g] | 0.120 (0.021) | 0.121 (0.025) | 0.825 |
| RMS combined planes (accelerometer) [g] | 0.166 (0.031) | 0.166 (0.035) | 0.975 |
| Mid-phase RMS ML (accelerometer) [g] | 0.117 (0.034) | 0.120 (0.038) | 0.697 |
| Mid-phase RMS combined planes (accelerometer) [g] | 0.15 (0.04) | 0.15 (0.04) | 0.990 |
| RMS VT (gyroscope) [g] | 1.10 (0.33) | 1.11 (0.28) | 0.867 |
| **Smoothness** |  |  |  |
| Jerk Max AP (accelerometer) [g/second] | 8.6 (3.4) | 10.8 (4.8) | 0.031 |
| Jerk Min ML (gyroscope) ^log10-reflect^ [g/second] | -19 (12) | -23 (13) | 0.070 |
| **Factor scores** |  |  |  |
| Initiation | -0.17 (0.96) | -0.20 (1.06) | 0.973 |
| Magnitude ^log10^ | -0.32 (0.60) | -0.05 (0.97) | 0.009 |
| Smoothness ^log10^ | 0.25 (0.86) | -0.05(1.06) | 0.209 |

Data displayed as mean (SD). Positively skewed variables were log10-transformed (denoted *log10*) square root-transformed (demoted *sqrt;* depending on the degree and nature of skewness). Negatively skewed variables were reflected and then log10-transformed (denoted *log10-reflect*), where reflection used y = log10(K-x) with K=max(x)+1. Variables that were approximately normal were analyzed on the raw scale (no notation). Bonferroni-adjusted significance threshold of *p* < 0.003. RMS: root mean square; VT: vertical; ML: mediolateral; AP: anterior-posterior.

**Supplemental Figure 3.** Boxplots demonstrating distribution of data for turning variables across cognitive impairment and control groups.


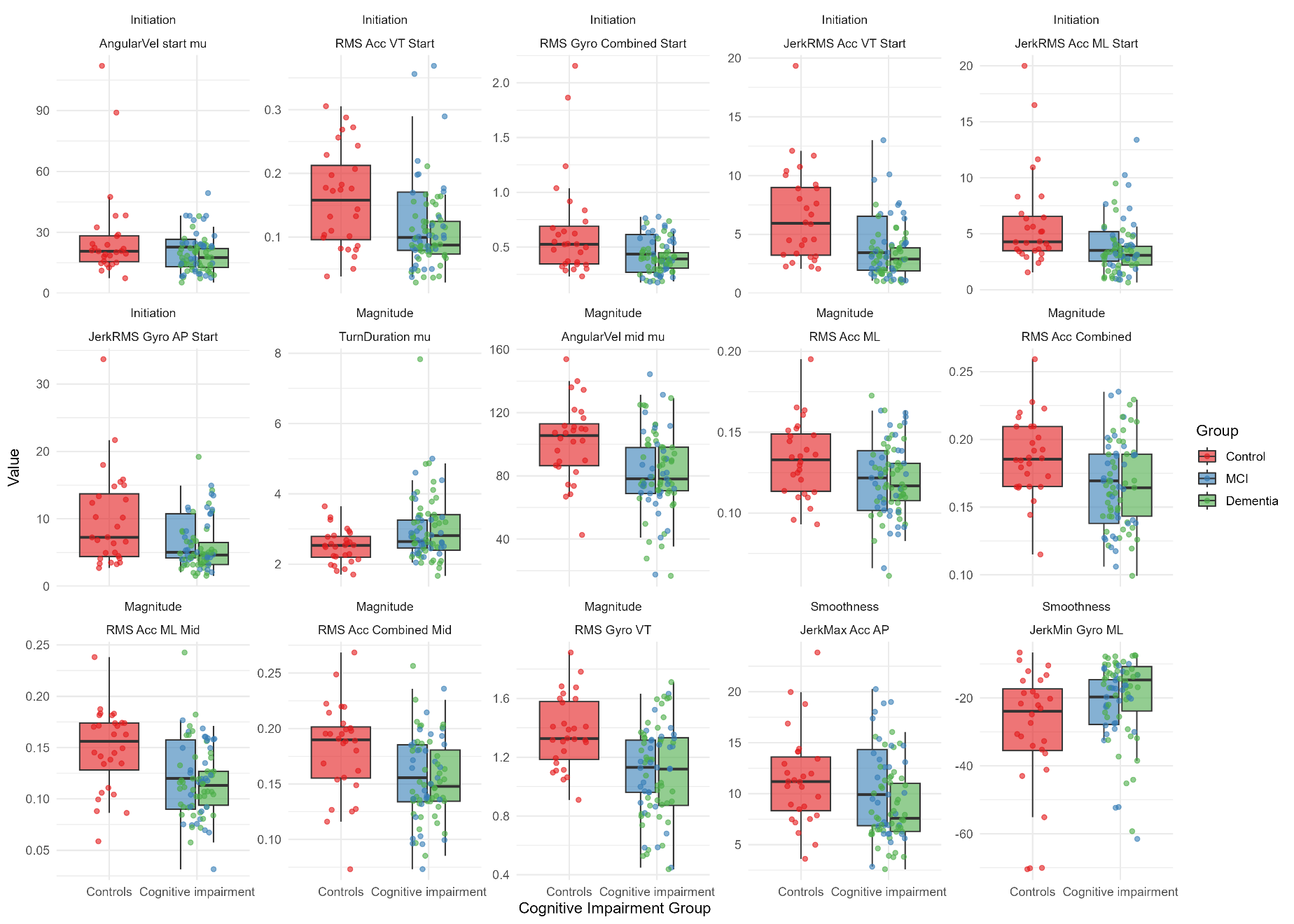


**Supplemental Table 5.** Between group comparisons for turning variables in cognitively impaired and control groups, with Parkinson’s disease cognitive impairment removed.

|  | **Cognitive Impairment**  N = 67 | **Cognitively Intact Older Adults** N = 28 | **p** |
| --- | --- | --- | --- |
| **Initiation** |  |  |  |
| Start-phase angular velocity^log10^ [degrees/second] | 20 (9) | 27 (23) | 0.147 |
| Start-phase RMS VT (accelerometer) ^log10^ [g] | 0.11 (0.07) | 0.16 (0.08) | **0.002** |
| Start-phase RMS combined (gyroscope) ^log10^ [g] | 0.42 (0.16) | 0.65 (0.46) | 0.010 |
| Start-phase Jerk RMS VT (accelerometer) ^log10^ [g/second] | 3.63 (2.40) | 6.62 (4.03) | **<0.001** |
| Start-phase Jerk RMS ML (accelerometer) ^log10^ [g/second] | 3.77 (2.45) | 5.98 (4.24) | **<0.001** |
| Start-phase Jerk RMS AP (gyroscope) ^log10^ [g/second] | 6.2 (3.8) | 9.8 (7.0) | 0.006 |
| **Magnitude** |  |  |  |
| Turn duration ^log10^ [seconds] | 2.94 (0.91) | 2.51 (0.48) | 0.085 |
| Mid-phase angular velocity [degrees/second] | 84 (24) | 102 (25) | 0.010 |
| RMS ML (accelerometer) [g] | 0.122 (0.023) | 0.133 (0.024) | 0.033 |
| RMS combined planes (accelerometer) [g] | 0.17 (0.03) | 0.19 (0.03) | 0.005 |
| Mid-phase RMS ML (accelerometer) [g] | 0.12 (0.04) | 0.15 (0.04) | **0.001** |
| Mid-phase RMS combined planes (accelerometer) [g] | 0.16 (0.04) | 0.18 (0.04) | 0.006 |
| RMS VT (gyroscope) [g] | 1.14 (0.30) | 1.37 (0.24) | 0.005 |
| **Smoothness** |  |  |  |
| Jerk Max AP (accelerometer) ^log10^ [g/second] | 9.5 (4.3) | 11.4 (4.6) | 0.021 |
| Jerk Min ML (gyroscope) ^log10-reflect^ [g/second] | -21 (13) | -30 (18) | 0.017 |
| **Factor Scores** |  |  |  |
| Initiation | -0.10 (0.98) | 0.50 (0.84) | 0.014 |
| Magnitude ^sqrt^ | -0.18 (0.81) | 0.57 (1.30) | 0.067 |
| Smoothness ^log10-reflect^ | 0.12 (0.97) | -0.35 (1.07) | 0.831 |

Data displayed as mean (SD). Positively skewed variables were square root (sqrt) or log10-transformed (denoted *log10*). Negatively skewed variables were reflected and then log10-transformed (denoted *log10-reflect*), where reflection used y = log10(K-x) with K=max(x)+1.Variables that were approximately normal were analyzed on the raw scale (no notation). Bolded values reflect those which meet statistical significance using the Bonferroni-adjusted significance threshold of *p* < 0.003. Color coding: Green boxes relate to large effect sizes, orange medium effect sizes and red small effect sizes. η^2^: eta squared; RMS: root mean square; VT: vertical; ML: mediolateral; AP: anterior-posterior.
